# College and Hospital Note

**Published:** 1910-02

**Authors:** 


### COLLEGE AND HOSPITAL NOTE.

The Buffalo Hahnemann Hospital to be erected on Lafayette Avenue is making a money campaign under the direction of George B. Montgomery, Esq., the purpose of which is to raise $150,000 in subscriptions. The time limit fixed upon was January 25, 1910. If, however, the desired sum should not be raised by that date, no doubt the time will be extended. Perhaps some kind friend will subscribe the balance needed thus making it unnecessary to extend the time. The cause is worthy the effort being made and every person able to do so should aid to the extent of his ability in this hospital building.

